# Low back pain in adolescent and associated factors: A cross sectional study with schoolchildren

**DOI:** 10.1590/bjpt-rbf.2014.0051

**Published:** 2014

**Authors:** Mônica R. O. G. C. M. Silva, Ana Fátima V. Badaró, Marinel M. Dall'Agnol

**Affiliations:** 1 Centro de Ciências da Saúde (CCS), Universidade Federal de Santa Maria (UFSM), Santa Maria, RS, Brazil; 2 Departamento de Fisioterapia e Reabilitação, CCS, UFSM, Santa Maria, RS, Brazil; 3 Departamento de Saúde da Comunidade, CCS, UFSM, Santa Maria, RS, Brazil

**Keywords:** low back pain, prevalence, epidemiology, adolescent, rehabilitation

## Abstract

**Objective:**

To determine the prevalence of low back pain nonspecific and associated factors in schoolchildren.

**Method:**

This cross-sectional study investigated 343 adolescents, aged between 12 and 15 years, of both sexes of public schools. The questionnaire included questions regarding sociodemographic characteristics, type of school transportation, body mass index and low back pain. The outcome was defined as discomfort localized below the costal margin and above the inferior gluteal folds in the last 12 months.

**Results:**

: The prevalence of low back pain in the last year was 57% (n=195) among participants, with no significant difference between the sexes (OR 1.13, 95% CI 0.93 to 1.37). Advancing age and body mass index were associated with the presence of low back pain in the bivariate analysis. The remaining seated at school in usual days was considered one of the main activities that trigger symptoms that lasted up to seven days for the majority (80%) of adolescents.

**Conclusions:**

The high prevalence of low back pain presented, indicating that it is a common condition among these adolescents. There was no difference between the sexes, but had influence of age and body mass index. Our results point to the need for the development epidemiological studies of low back pain among children and adolescents.

## Introduction

Low back pain (LBP) or lumbago is characterized by pain or discomfort in the lumbar region, below the costal margin and above the gluteal fold that may or may not irradiate to the thigh^1^. LBP is a sensory and emotional experience that may be associated with trauma. This condition is difficult to diagnose^2^, since many different factors (e.g. physiological, emotional and cultural) are responsible for triggering pain stimuli in individuals. Due to the complaints being subjective, it is challenging to characterize and to describe this multidimensional experience, or to quantify it in numbers or measurable data^3^.

The diagnostic of LBP, obtained through the clinical history of the patient associated with a physical examination, classifies LBP into three categories: 1) LBP potentially associated with a specific cause in the spine, 2) LBP potentially associated with spinal stenosis, or 3) LBP with a nonspecific cause^1^. The first two diagnoses have defined etiology, since the pain has a specific cause (i.e. congenital, neoplastic, inflammatory, infectious, metabolic, traumatic, degenerative or functional) and this pain affects less than 15% of the adult, adolescent and childhood populations^4^. On the other hand, for the nonspecific LBP, the causal agent is unknown^5^.

In Europe, the cumulative annual incidence of LBP symptoms in adolescents is 24%^6^, with prevalence reaching more than half of these individuals^7^^,^ 8. It has become a common condition among adolescents, similar to adults, and may reach 70-80% of the population under 20 years of age^9^. Several investigations on LBP in children and adolescents have been conducted in other countries^7^^-^ 9, but few studies have investigated the symptoms within this age group in Brazil^10^^-^ 12. Furthermore, there is a great difference between the prevalence rates in Brazilian studies, ranging from 13% to 49% in individuals between 11 and 19 years of age^11^^,^ 12.

The literature has shown that, when considering the etiology of the nonspecific LBP, the possible risk factors include: age^13^^,^ 14, female gender^13^^,^ 14, race/ethnicity^1^, type of transportation used to go to school^11^, tight muscles^15^, accelerated growth in height^15^^,^ 16, remaining seated for long hours^17^, child work^17^^,^ 18, psychosocial barriers^19^ and body mass index (BMI)^20^. There are authors who also considered low parental educational status, which is an indicator of socio economic status of the family, as a factor that has been associated with LBP in children and adolescents^21^.

Similar to what occurs among adults^22^, LBP might affect the social lives of adolescents at school and during leisure^23^^,^ 24, as well as their economic life, since the condition triggers costs for doctors and treatments. Therefore, an investigation of the prevalence of LBP and its risk factors are important for the development of preventive measures^5^ and effective interventions to improve the quality of life of these young people.

The present study had the objective of determining the prevalence of LBP among adolescents in schools in southern Brazil, and its association with socio demographic factors, types of school transportation and BMI.

## Method

This cross-sectional study evaluated students from the 6^th^ to the 9^th^ year of elementary education in public schools, 12-15 years of age, that were living in the city of Santa Maria, RS, Brazil. The Municipal Secretary of Education authorized the project and provided a list of 45 elementary public schools in the urban area and the number of students per grade, by school and by administrative region. A total of 5,471 students were enrolled in 2012.

The sample size a power calculation was made using *EpiInfo(tm) software version 6.04*. The aim was to have 80% power to observe a significant diference (alpha level) of 5%, with prevalence ratio of 1.3 and estimated dropout rate of 20%, we needed to include 328 adolescents in group.

The sampling was performed in multiple stages, with stratified cluster selection proportional to the number of students enrolled in each of the six urban administrative regions of the municipality. One school per region was selected by simple random sampling, and all students of the target population were invited to participate. When there was refusal of the school to participate in the study, another school was drawn until the sample reached the number of students required. One school refused to participate in the study on the grounds that the data collection would delay the school schedule and a second school because of the existence of other health projects that were already in development. Therefore two other schools were selected to make up the final sample.

Exclusion criteria were students with alterations in the central nervous system (CNS) (e.g. cerebral palsy, paraplegia) and/or cognitive deficits, based on the school records; students with musculoskeletal problems (e.g. fractures in the upper limbs, lower limbs, trunk or a prosthesis) and pregnancy, as these conditions could interfere with the quality of the anthropometric data.

The project was approved by the Ethical Committee in Research from Universidade Federal de Santa Maria (UFSM), Santa Maria, RS, Brazil, under protocol number 7061/2012. The researcher instructed the students about the informed consent (IC), so that parents/or guardians could authorize their participation in the study. Likewise, the adolescents also signed a consent form.

Data collection was carried out between May and July 2012 during two visits: one for the application of the questionnaire and another for the measurement of the body weight and height, both performed by one of the researchers. The adolescents completed the questionnaire after the interviewer had read each question and its alternatives, had clarified any confusion and had emphasized that the answers were independently given. The interview took place in a room reserved by the school, with a group of 20 to 25 students, and lasted approximately 30 minutes for each student. The questions were pre-coded and addressed socio demographic characteristics, and types of transportation used to go to school, the presence of LBP and body positions that would trigger the pain. In addition, the measurements of body weight and height were obtained to calculate the BMI.

The socio demographic variables included age, gender, paid child work (yes or no), self-chosen ethnic group (i.e., Caucasian, Black, Brown, Asian and Indigenous) and the highest level of education of the parent/guardian responsible for the adolescent (i.e., no education, incomplete or complete primary education, incomplete or complete secondary education, incomplete or complete post-secondary education).

The type of transportation used to go to school was classified as: on foot, by bicycle, by bus, by car or other. The responses were grouped into "passive transportation" when the adolescent travelled by car, motorcycle or bus; and "active transportation" when they travelled on foot or by bicycle. The type of transportation, the colour of their skin and the parents' level of education were grouped for the bivariate analysis, forming one of the many categories presented in the tables to facilitate the tests.

The dependent variable, nonspecific LBP, was anatomically defined as any pain that occurred between the twelfth rib and the inferior gluteal fold without radiation to the lower limbs^1^^,^ 5. To increase the accuracy of data collection, we added the following information, a minimum duration of 24 hours of symptoms since onset, to avoid reports of LBP due to fatigue, which can be resolved within a few hours^19^. To measure this outcome we used the adapted version of the Nordic Questionnaire (Standardized Nordic Questionnaire, originally created by Kuorinka^25^) proposed by Sjolie^26^, for children and adolescents. The Brazilian Portuguese version was validated and showed an excellent intraclass correlation coefficient (0.70 to 0.99) and Cronbach's alpha (0.795). The questionnaire showed the lower back region in a drawing of the human body and investigated pain through nine dichotomous answers (yes or no). The instrument evaluated LBP reports within the last year and its duration in days (i.e., one to seven days, eight to 30 days, more than 30 days - but not every day, and every day) as well as the presence of trauma in the lumbar spine (for exclusion criteria). In addition, it evaluated the respondent's ability to recognize whether or not the pain was triggered or enhanced by certain body positions (i.e., seated in school, seated at home, seated in a car, seated in situations other than those already listed, watching television, or physical work)^14^.

The BMI, obtained by dividing the body mass in kilograms by the square of the height in meters, was classified as follows: excessively lean if less than 17 Kg/m^2^, normal if between 17 and 24.9 Kg/m^2^, overweight if between 25 and 29.9 Kg/m^2^ and obese if ≥30 Kg/m^2^. This classification is considered the most appropriate for children and adolescents^27^. The overweight and obese categories were grouped into the category "overweight" for analysis. Body mass and height were recorded using an electronic digital scale (Soehnle brand) with a stadiometer consisting of a metal rod scaled from zero to 2.30 meters and with intervals of one centimeter. These measurements were recorded using the techniques recommended by the World Organization of Health^28^.

The information was stored in a database in EpiInfo 6.04, with double entry and data validation. This was followed by descriptive and bivariate analyses. The descriptive analysis showed the distributions of absolute and relative frequencies for categorical variables. For the continuous variables, mean and standard deviation were applied. For analysis, the bivariate analysis with calculations of Odds Ratio (OD) with the 95% confidence interval (CI), and the chi-square for nominal variables, were conducted. Associations with p≤0.05 were considered significant.

## Results

This cross-sectional study assessed 355 adolescents attending public schools in 2012. Sample loss from those who agreed to participate in the survey accounted for 3% of the total sample (n=12), and occurred due to the interruption of the interview process by a professor who had scheduled other activities for the students. The final sample included 343 individuals, with a greater number of girls, caucasian or brown, normal BMI and with a mean age of 13 years (SD=0.96) (Table 1). Almost half of the parents interviewed had an educational level lower than elementary school education; most students walked to school, and the paid child labour was observed in 13%. These characteristics are shown in Table 2.

**Table 1:** Sample distribution according to gender, age, ethnic group and BMI of adolescents from the public schools in Santa Maria, RS, Brazil, 2012 (n=343).

	n	%
Gender		
Female	210	61%
Male	133	39%
Age Group		
12 years	117	35%
13 years	119	36%
14 years	67	20%
15 years	32	10%
Ethnic Group		
Caucasian	164	49%
Black	41	12%
Brown	114	34%
Indigenous	15	5%
Asian	3	1%
BMI		
Excessive lean	39	12%
Normal	236	72%
Overweight	41	13%
Obese	10	3%

Until 2% unrecognized answer and losses in the questionnaire; 4-5% unrecognized answer and losses in the questionnaire.

**Table 2:** Sample distribution according to parental educational level, childhood paid labour and school transportation used by adolescents from public schools in Santa Maria, RS, Brazil, 2012 (n=343).

	n	%
Parental educational level		
None	8	3%
Elementary school (incomplete)	124	43%
Elementary school (complete)	27	9%
High School (incomplete)	65	23%
High School (complete)	63	22%
Childhood paid labour		
Yes	44	13%
No	297	87%
School transportation used		
On foot	276	84%
Bus	24	7%
Other	14	4%
Car	11	3%
Bike	3	1%

Until 2% unrecognized answer and losses in the questionnaire; 4-5% unrecognized answer and losses in the questionnaire; 16% unrecognized answer and losses in the questionnaire.

For the adolescents who reported back pain in the last year, the most reported activity and/or body position that triggered or increased pain was seated in school followed by seated in another situation, seated at home, doing physical work, physical education classes, watching TV and seated in the car, as shown in Table 3.

**Table 3:** Activities or triggering positions that aggravated low back pain in adolescents from public schools in Santa Maria, RS, Brazil, 2012 (n=195).

	n	%
Seated at school	131	70%
Seated in other situation	108	60%
Seated at home	102	57%
Heavy job	98	53%
Physical education classes	80	45%
Watching TV	60	33%
Seated in the car	30	17%

4-8% unrecognized answer and losses in the questionnaire.

The prevalence of LBP in the last year was 57% (n=195) among participants, 60% in girls (n=125) and 53% in boys (n=70), with no significant difference between the sexes (RP=1.13, 95% CI 0.93 to 1.37). The vicariate analysis also showed a trend of increased prevalence of LBP in older adolescents, with a statistically significant difference between 14 year old students compared to 12 year olds.

The BMI was associated with LBP and the prevalence was significantly lower among excessively lean students compared to normal individuals. There was no difference in LBP between obese compared with students having a normal weight. Their ethnic group (Table 4), doing paid child labour, level of parental schooling and type of transportation to school were not associated with LBP (Table 5).

**Table 4:** Bivariate analysis of the prevalence of low back pain according to gender, age, ethnic group and BMI of adolescents from public schools in Santa Maria, RS, Brazil, 2012 (n=343).

	n	Low back pain prevalence	OR (95%CI)	p-value
Gender				
Female	125	60%	1.13 (0.93-1.37)	0.21
Male	70	53%	1.00	
Age				
12 years	59	51%	1.00	
13 years	66	55%	1.09 (0.86-1.39)	0.48
14 years	44	67%	1.31 (1.02-1.68)	0.039
15 years	20	63%	1.23 (0.89-1.70)	0.24
Ethnic Group				
Caucasian	93	43%	0.96 (0.83-1.21)	0.99
Other	97	43%	1.00	
BMI				
Excessive lean	15	39%	0.66 (0.43-0.99)	0.01
Normal	138	59%	1.00	
Over weight or obese	34	67%	1.14 (0.91-1.42)	0.29

OR: odds ratio; CI: confidence interval; p-value <0.05.

**Table 5:** Bivariate analysis of the prevalence of low back pain, according to parental educational level, paid childhood labour, school transportation used for adolescents from public schools in Santa Maria, RS, Brazil, 2012 (n=343).

	n	Low back pain prevalence	OR (95%CI)	p-value
Parental educational level				
Elementary school or lower	130	58%	1.10 (0.85-1.42)	0.47
High school or higher	33	53%	1.00	
Childhood labour paid				
Yes	27	61%	1.08 (0.84-1.4)	0.55
No	167	57%	1.00	
School transport used				
Active transport	170	58%	1.20 (0.84-1.71)	0.27
Passive transport	17	49%	1.00	

OR: odds ratio; CI: confidence interval; p Value <0.05.

The symptoms of LBP lasted up to seven days for most students who reported pain (80%), but 7% reported that pain was present every day and 5% of those students had pain that lasted longer than 30 days. There was no association between the duration of LBP and other variables assessed.

## Discussion

This study found that LBP was highly prevalent in students 12 to 15 years of age from public schools. In addition, the prevalence was similar among boys and girls. The cross-sectional design of this study prevented inference of causality, however, the sample size was sufficient to detect relationships between the outcome and the variables studied. Being older was associated with increased occurrence of low back pain, however, this occurrence was less frequent among excessively lean adolescents (i.e., those with BMI <17Kg/m^2)^.

Studies on the prevalence of LBP among Brazilian adolescents are scarce^10^^-^ 12. Furthermore, the data presented regarding the definition of LBP and the types of instruments used to collect data are conflicting. To minimize these issues, our study followed the standard definition of LBP established by the European Guidelines for Prevention of Low Back Pain (*European Guidelines for Prevention in Low Back Pain*) by the group *Cost Action B13* 5. This research group was created by the European Commission to establish guidelines for the management of LBP. It consisted of invited researchers, experts in the field of LBP, from nine countries. The instrument chosen to determine the outcome was adapted to the population of interest and has been shown to have good reproducibility^14^.

Based on the criteria measured, this study observed a higher prevalence of LBP than the study of Vitta (19%) conducted in the southeast of Brazil^10^. The findings were similar to international reports, demonstrating that LBP is a common condition among adolescents^7^^,^ 8 ^,^ 25, as well as among adults^22^. Although measures were taken to ensure subject participation, some students refused to participate in this investigation. This result is similar to other studies conducted with adolescents using questionnaires^15^^,^ 29 ^,^ 30. However, the observed high prevalence suggests that the non-participants did not affect the study's outcome. It also suggests that the outcome was not underestimated and the expected random distribution of the non-respondents did occur. In addition, the two variables observed, age and BMI, were associated with non-specific LBP.

Age showed a direct relationship with LBP, a fact that has been supported by most studies^16^^,^ 18 ^,^ 19 ^,^ 24 ^,^ 29, and according to some researchers, strongly associated with the growth spurt observed in adolescence^15^^,^ 16. The peak of the growth spurt, which occurs earlier for girls (on average between the ages of 11 and 12 years) than for boys (commonly between the ages of 13 and 14)^31^, may be responsible for the decreased flexibility, especially of the quadriceps and hamstrings muscles^15^ , that causes functional impairment of the lumbar muscles, and consequently pain.

In this study, LBP was commonly seen in the 14 year old group. It is possible that the small number of 15 year old participants in the sample (10%), compared with the 14 year old (20%), may have influenced the results, despite the fact that LBP has been shown to be prevalent among 15 year old adolescents.

Even with the high occurrence of overweight and obese adolescents (16%), the findings of the relationship of LBP and BMI were inconsistent with the findings of a previous study^20^^,^ 32 ^,^ 33, since excess body weight was not correlated with LBP in the present study, even with the slight increase as compared to normal. Because most of the studies^20^^,^ 32 ^,^ 33 are generally focused on excess body weight, it is necessary to evaluate the influence of the non-respondents, but it was not possible to carry out this procedure, in the present study.

On the other hand, the low occurrence of LBP among excessively lean adolescents is of note. In this sample, a BMI<17 Kg/m^2^ appears to be a factor that decreases the occurrence LBP. However, this finding has not been emphasized by the literature reviewed, because the focus of most of the studies^20^^,^ 32 ^,^ 33 have generally involved the overweight group. According to our results, it is important to consider that the assessments based on BMI could be a complicating factor due to the changes in body composition that occur during sexual maturation of the adolescent^34^.

In this study, the seated position during the usual school day was one of the activities considered harmful to the lumbar region, both for those who had LBP and those who did not. It is possible that seated postures^12^^,^ 17 and school furniture^7^ might lead to lumbar muscle dysfunction and predispose the individual to painful symptoms^12^^,^ 16 ^,^ 17. However, this conjecture was not assessed in the present study.

No association was found between LBP and gender in this study, which it is consistent with some of the other studies^6^^,^ 29 in the literature. Nevertheless, there is no consensus regarding this gender outcome, since there are authors who have found an association between LBP and females^7^^,^ 8 ^,^ 10 ^,^ 17 ^,^ 19, and others with males^16^^,^ 29. Furthermore, other variables (ethnicity, type of school transport, education level of responsibility and child work) were not significantly associated with LBP. However, the literature indicates some of these variables as risk factors for the presence of painful symptoms in the lumbar region in schoolchildren^11^^,^ 13 ^,^ 18 ^-^ 20.

This study showed that there is evidence that problems in the lumbar spine in children and adolescents are common, but more epidemiological studies should be performed to assess the prevalence and incidence in other geographical regions of Brazil, which may have cultural, social and climate differences from the one studied. In addition, other factors need to be investigated. For example: do the symptoms persist over a number of years? do the majority of children with these problems receive appropriate treatment? do untreated disorders lead to the occurrence of severe LBP in adulthood? do the symptoms limit activities? what is the impact of this severity on health services? Finally, how does this condition affect the quality of life of young people?

## Conclusion

The knowledge of the prevalence and incidence of LBP in a specific population, as well as the identification of the risk factors, forms the basis for the development of treatments and prevention programs and assists in the planning of health care services directed to help children and adolescents. In the present study, LBP showed a high prevalence in the 12 to 15 year old age group, indicating that it is a common condition among adolescents. The majority of the participants reported that the symptoms lasted for a week, were similar among boys and girls, were significantly higher in older adolescents, and were lower among the excessively lean students. In addition, the seated position during school days was considered the main activity that triggered LBP symptoms.

## References

[1] Chou R, Qaseem A, Snow V, Casey D, Cross JRT, Shekelle P (2007). Diagnosis and treatment of low back pain: a joint
clinical practice Guideline from the American College of Physicians and the
American Pain Society. Ann Intern Med.

[2] International Association for the Study of Pain - IASP (2006). Psychological intervention for acute and chronic pain in
children. Pain.

[3] Silva JA, Ribeiro-Filho NP (2011). A dor como um problema psicofísico. Rev Dor.

[4] Brazil A, Ximenes AC, Radu AS, Fernandes AR, Appel C, Maçaneiro CH (2004). Diagnóstico e tratamento das lombalgias e
lombociatalgias. Rev Bras Reumatol.

[5] Burton AK, Balagué F, Cardon G, Eriksen HR, Henrotin Y, Lahad A (2006). Chapter 2. European guidelines for prevention in low
back pain. Eur Spine J.

[6] Jones GT, Watson KD, Silman AJ, Symmons DPM, Macfarlane GJ (2003). Predictors of low back pain in British schoolchildren: a
population-based prospective cohort study. Pediatrics.

[7] Harreby MS, Nygaard B, Jessen TT, Larsen E, Storr-Paulsen A, Lindahl A (2001). Risk factors for low back pain among 1.389 pupils in the
8th and 9th grade: an epidemiologic study. Ugeskr. Laeg.

[8] Kovacs FM, Gestoso M, Gil Del Real MT, López J, Mufraggi N, Méndez JI (2003). Risk factors for non-specific low back pain in
schoolchildren and their parents: a population based study. Pain.

[9] Jeffries LJ, Milanese SF, Grimmer-Somers KA (2007). Epidemiology of adolescent spinal pain: a systematic
overview of the research literature. Spine (Phila Pa 1976).

[10] De Vitta A, Martinez MG, Piza NT, Simeão SFAP, Ferreira NP (2011). Prevalência e fatores associados à dor lombar em
escolares. Cad. Saúde Pública.

[11] Onofrio AC (2010). Dor lombar aguda em adolescentes do ensino médio de uma cidade
do sul do Brasil: prevalência e fatores associados [dissertation].

[12] Graup S, Santos SG, Moro ARP (2010). Estudo descritivo das alterações posturais sagitais da
coluna lombar em escolares da rede federal de ensino de
Florianópolis. Rev Bras Ortop.

[13] Watson KD, Papageorgiou AC, Jones GT, Taylor S, Symmons DP, Silman AJ (2003). Low back pain in schoolchildren: the role of mechanical
and psychosocial factors. Arch Dis Child.

[14] Vidal ARC (2009). Dor lombar específica em alunos adolescentes em função do
gênero, idade e nível de atividade física [master's thesis].

[15] Feldman DE, Shier I, Rossignol M, Abenhaim L (2001). Risk factors for the development of low back pain in
adolescence. Am J Epidemiol.

[16] Poussa MS, Heliövaara MM, Seitsamo JT, Könönen MH, Hurmerinta KA, Nissinen MJ (2005). Anthropometric measurements and growth as predictors of
low-back pain: a cohort study of children followed up from the age of 11 a 22
years. Eur Spine J.

[17] Sjolie AN (2004). Persistence and changes in nonspecific low back pain
among adolescents. Spine (Phila Pa 1976).

[18] Fassa AG, Facchini LA, Dall'Agnol MM, Christiani DC (2005). Child labor and musculoskeletal disorders: the Pelotas
(Brazil) epidemiological survey. Public Health Rep.

[19] Jones GT, Macfarlane GJ (2009). Predicting persistent low back pain in schoolchildren: a
prospective cohort study. Arthritis Rheum.

[20] Shiri R, Karppinen J, Leino-Arjas P, Solovieva S, Viikari-Juntura E (2010). The association between obesity and low back pain: a
meta-analysis. Am J Epidemiol.

[21] Hestbaek L, Korsholm L, Leboeuf-Yde C, Kyvik KO (2008). Does socioeconomic status in adolescence predict low
back pain in adulthood? A repeated cross-sectional of 4,771 Danish
adolescents. Eur Spine J.

[22] Ferreira GD, Silva MC, Rombaldi AJ, Wrege ED, Siqueira FV, Hallal PC (2011). Prevalence and associated factors of back pain in adults
from southern Brazil: a population-based study. Rev Bras Fisioter.

[23] Skoffer B, Foldspang A (2008). Physical activity and low-back pain in
schoolchildren. Eur Spine J.

[24] Prista A, Balangué F, Nordin M, Skovrom ML (2004). Low back pain in mozambican adolescents. Eur Spine J.

[25] Kuorinka I, Jonsson B, Kilbom A, Vinterberg H, Biering-Sorensen F, Andersson G (1987). Standardised Nordic questionnaires for the analysis of
musculoskeletal symptoms. Appl Ergon.

[26] Sjolie AN (2003). Active or passive journeys and low back pain in
adolescents. Eur Spine J.

[27] Cole TJ, Belizzi MC, Flegal KM, Dietz WH (2000). Establishing a standard definition for child overweight
and obesity worldwide: international survey. BMJ.

[28] World Health Organization - WHO (1995). Physical status: the use and interpretation of
anthropometry.

[29] Hestbaek L, Leboeuf-Yde C, Kyvik KO, Manniche C (2006). The course of low back pain from adolescence to
adulthood: eight-year follow-up of 9600 twins. Spine (Phila Pa 1976).

[30] Mikkelsson LO, Nupponen H, Kaprio J, Kautiainen H, Mikkelsson M, Kujala UM (2006). Adolescent flexibility, endurance strength, and physical
activity as predictors of adult tension neck, low back pain, and knee injury: a 25
year follow up study. Br J Sport Med.

[31] Instituto Brasileiro de Geografia e Estatística - IBGE (2009). Pesquisa Nacional de Saúde do Escolar: PeNSE.

[32] Auvinen JP, Tammelin TH, Taimela SP, Zitting PJ, Järvelin M, Taanila AM (2009). Is insufficient quantity and quality of sleep a risk
factor for neck, shoulder and low back pain? A longitudinal study among
adolescents. Eur Spine J.

[33] Balangué F, Bibbo E, Mélot C, Szpalski M, Gunzburg R, Keller TS (2010). The association between isoinertial trunk muscle
performance and low back pain in male adolescents. Eur Spine J.

[34] Conde WL, Monteiro CA (2006). Body mass index cut off points for evaluation of
nutritional status in Brazilian children and adolescents. J Pediatr.

